# Navigating Choices: Determinants and Outcomes of Surgery Refusal in Thyroid Cancer Patients Using SEER Data

**DOI:** 10.3390/cancers15143699

**Published:** 2023-07-20

**Authors:** Mohammad H. Hussein, Eman A. Toraih, Ifidon E. Ohiomah, Nabeela Siddeeque, Marie Comeaux, Madeleine B. Landau, Allison Anker, Jessan A. Jishu, Manal S. Fawzy, Emad Kandil

**Affiliations:** 1 Division of Endocrine and Oncologic Surgery, Department of Surgery, School of Medicine, Tulane University, New Orleans, LA 70112, USA; mhussein1@tulane.edu; 2 Genetics Unit, Department of Histology and Cell Biology, Faculty of Medicine, Suez Canal University, Ismailia 41522, Egypt; 3 School of Medicine, Tulane University, New Orleans, LA 70112, USA; iohiomah@tulane.edu (I.E.O.); nsiddeeque@tulane.edu (N.S.); mcomeaux2@tulane.edu (M.C.); mlandau@tulane.edu (M.B.L.); aanker@tulane.edu (A.A.); jjishu@tulane.edu (J.A.J.); 4 Department of Medical Biochemistry and Molecular Biology, Faculty of Medicine, Suez Canal University, Ismailia 41522, Egypt; manal2_khashana@ymail.com; 5 Department of Biochemistry, Faculty of Medicine, Northern Border University, Arar 91431, Saudi Arabia

**Keywords:** thyroid cancer, surgery, refusal, delay, SEER

## Abstract

**Simple Summary:**

Thyroid cancer is the most common endocrine cancer to date, and the standard treatment involves some form of surgical intervention. Even in cases where the clinician explicitly recommends such intervention, patients may refuse to undergo these procedures, which may lead to unfavorable outcomes. This study will attempt to understand the factors contributing to a patient’s decision to refuse surgery. To observe the dichotomy between patients who refuse surgery and those who undergo it, we assessed the cancer-specific and overall mortality for this cohort. Furthermore, we looked at the effect of delayed surgery on treatment outcomes using the same parameters previously described.

**Abstract:**

With thyroid cancer being a prevalent endocrine cancer, timely management is essential to prevent malignancy and detrimental outcomes. Surgical intervention is a popular component of the treatment plan, yet patients often refuse to undergo such procedures even if clinicians explicitly recommend them. This study gathers data from the Surveillance, Epidemiology, and End Results database (2000–2019) to learn more about the sociodemographic factors that predict the likelihood of surgical intervention. A total of 176,472 patients diagnosed with either papillary or follicular thyroid cancer were recommended surgery, of which 470 were refused. Cancer-specific mortality and overall mortality were determined with the Kaplan–Meier method and univariate and multivariate Cox proportional hazards regression model. Mortality rates for patients who delayed surgery (≥4 months vs. <4 months) were determined using similar methods. The findings reveal that surgical delay or refusal increased overall mortality. The surgical refusal was associated with increased thyroid cancer-specific mortality. However, the impact on thyroid cancer-specific mortality for those who delay surgery was not as pronounced. Significant sociodemographic determinants of surgical refusal included age greater than or equal to 55 years, male sex, being unmarried, race of Asian and Pacific Islander, and advanced tumor staging. The results underscore the importance of patient education, shared decision-making, and access to surgical interventions to optimize outcomes in thyroid cancer management.

## 1. Introduction

Thyroid cancer (TC) is a recognized adversary in medicine and successful disease management often necessitates a multifaceted treatment plan. Different interventions, such as surgical resection, are essential in controlling TC as well as other malignancies and they often contribute to considerable improvements in patients’ overall survival (OS), disease-specific survival (DSS), and prognosis [1]. For example, a recent study analyzing margin positivity and survival outcomes of patients with papillary thyroid carcinoma revealed that lower positive margins were directly correlated with higher surgery rates [2]. However, refusing recommended surgical intervention can profoundly impact the disease trajectory and prognosis of those who take that path [3,4]. Further research has also revealed that a lobectomy does not have adverse long-term outcomes for patients with papillary thyroid carcinoma (PTC) [5], implying that safety doubts may not be a substantial reason for refusing surgery. Thus, understanding the dynamics behind refusal is crucial for healthcare providers who seek to tailor individualized, patient-centered treatment plans.

While surgical interventions are efficacious, several studies have consistently demonstrated that patients with cancer who refuse surgery tend to experience worse clinical outcomes compared to those who opt in. For example, a recent study based out of the Stanford University School of Medicine study utilizing the Surveillance, Epidemiology, and End Results (SEER) database reported a 91% survival rate after five years for patients who underwent cancer-directed surgery, while those who refused the procedure had a 23% survival rate [6]. Similar correlations were found in non-TC patients with other types of cancer, such as those involving the colon and liver [7,8]. While socioeconomic factors have been identified as contributors to refusing surgery, comprehensive studies on these factors remain limited and highlight the need for further exploration [9,10]. Clinical variables, such as tumor stage, may also impact a patient’s decision to undergo surgery. It is reasonable to suggest that a more advanced disease stage could engender feelings of futility regarding surgical intervention, potentially leading to refusal, which may pose challenges in treatment planning and exacerbate disease prognosis and recurrence risk, even while respecting patient autonomy [11]. As such, elucidating the determinants of refusing surgery among TC patients when it is recommended could provide valuable insight and assist in the development of personalized strategies aimed at optimizing patient–provider communication, improving decision-making processes, and overcoming access barriers.

In addition to refusing surgery, another critical factor that may impact TC outcomes is the timing of intervention. Delayed surgery has been highlighted as a potential risk factor for poor clinical outcomes and worse prognosis in various malignancies [12,13]. These findings emphasize timely surgical intervention in TC management and the need for active efforts to minimize delays in surgical care. Identifying the key determinants of delayed surgery, in addition to refusal, can enable healthcare providers to address these issues and improve access to healthcare, especially for patients facing significant socioeconomic barriers.

Overall, this study examines a large patient cohort over an extended period to explore the interplay of demographic, socioeconomic, and clinical factors in the decision to refuse surgical interventions using the SEER database. With this analysis, we hope to shed light on diverse factors influencing patients’ surgical decisions and provide insights to refine treatment strategies, improve access to care, and enhance the overall quality of cancer management. Our goal is to contribute to improving patient outcomes and offering patient-centered care. By presenting our findings, we hope to stimulate further research into TC care and provide a deeper understanding of the decision-making processes involved in cancer management.

## 2. Materials and Methods

### 2.1. Data Source

This retrospective cohort study utilized data from the Surveillance, Epidemiology, and End Results (SEER) database (Registry 17) to investigate the factors associated with patients refusing recommended cancer surgery. The SEER database is a nationally representative source that includes patients diagnosed with papillary (PTC) and follicular thyroid cancer (FTC) who were recommended surgery. The study period spanned from 2000 to 2019. Tulane University waived ethical approval.

### 2.2. Study Population and Variables

The initial pool of patients diagnosed with TC in the SEER registry was determined to be 203,728. After excluding patients diagnosed incidentally at autopsy and those identified via death certificates, a subset of 176,472 patients with positive histological confirmation of cancer was identified. This subset excluded anaplastic thyroid cancer (ATC) and medullary thyroid cancer (MTC). Patients missing survival or surgery data were excluded from this study. The final dataset included 166,311 papillary thyroid cancer (PTC) diagnoses and 10,161 follicular thyroid cancer (FTC) diagnoses. The examination parameters included a wide array of demographic, clinical, and therapeutic factors such as age, sex, racial background, Hispanic/Latino ethnicity, urban or rural habitation, annual household income, histopathological subtype, previous malignancies, tumor size, tumor-node-metastasis (TNM) staging according to the AJCC 8th system, and the extension of cancer. Factors such as surgical interventions targeting cancer and radiation therapy were also assessed.

### 2.3. Primary Outcomes

The study aimed to provide a comprehensive understanding of the outcomes of patients who refused recommended surgery and those who underwent surgery for FTC and PTC diagnoses. The study examined the survival rate, cancer recurrence rate, and the chances of second primary malignancy based on different cancer stages (I, II, III, or IV) for patients who either underwent surgery (*n* = 176,002) or refused recommended surgery (*n* = 470). Next, after removing missing observations, patients with delayed surgery ≥4 months (*n* = 5257) were compared to those with earlier intervention (<4 months) (*n* = 140,149). Disease outcomes, including the possibilities of cancer recurrence, second primary malignancy, and survival status, were also analyzed.

### 2.4. Statistical Analysis

The statistical analyses were performed using the R statistical language (version 4.2.2; R Core Team, 2022) on macOS Ventura 13.3.1 and SPSS version 27.0 (IBM Corp., Armonk, NY, USA). A significance level of 0.05 was used for all analyses and all tests were two-sided. Categorical variables were presented as frequencies and percentages, while continuous variables were reported as mean (standard deviation) or median (interquartile range), as appropriate. Descriptive statistics, such as Chi-square or Fisher’s exact tests for categorical variables and Student’s *t*-test or Mann–Whitney U test for continuous variables, were used. Logistic regression models were employed to identify independent predictor risk factors for recurrence and second primary malignancy, adjusting for potential confounders. Odds ratios (ORs) with 95% confidence intervals (CIs) were reported. TC-specific and overall survival analyses were conducted using the Kaplan–Meier method, and the log-rank test was used to assess differences in survival between groups. Prognostic factors for survival were identified using univariate and multivariate Cox proportional hazards regression models. Multivariate models were built using significant variables in the univariate analyses and hazard ratios (HRs) with 95% CIs were reported.

## 3. Results

### 3.1. Characteristics of the Study Population

The study encompasses a large sample size of 176,472 patients, collated from the SEER database (Registry 17: 2000–2019). Among them, a predominant majority, 99.7% or 176,002, underwent surgery, while a mere 0.3% or 470 individuals declined the procedure. Analyzing the demographic characteristics (Table 1), it is noteworthy that the median age for those who underwent surgery was 49.0 years (IQR 38–60 years), which was markedly lower than the median age of 59.0 years (IQR 44–74 years) among those who refused surgery, a statistically significant disparity (*p* < 0.001). Gender distribution was tilted towards females, who constituted 76.5% of the total patient cohort and the surgery group. Conversely, the proportion of males was noticeably higher in the refusal group (31.3%).

The racial composition was predominantly White (82.1%), followed by API (Asian or Pacific Islander) at 10.9%, Black at 6.3%, and AI/AN (American Indian/Alaska Native) at 0.7%. However, the refusal group demonstrated a higher proportion of API (19.5% versus 10.9% in the surgery group) and AI/AN patients (1.5% versus 0.7% in the surgery group, *p* < 0.001). Regarding ethnicity, most were identified as non-Hispanic/Latino (83.4%), with no discernible difference between the surgical and refusal groups (*p* = 0.68).

In the context of marital status, those who were married or in a domestic partnership formed the majority (64.8%). However, such individuals were less inclined to refuse surgery (48.9% versus 64.9% in the surgery group, *p* < 0.001). Of particular interest was the higher proportion of widowed individuals within the refusal group (15.9% versus 5.0% in the surgery group). Most of the patient cohort resided in metropolitan areas with populations exceeding 1 million (60.2%). Additionally, the most considerable income bracket among patients was USD 75,000 (34.4%). However, no statistically significant differences were observed between the surgical and refusal groups regarding residency and annual household income (*p* = 0.11 and 0.33, respectively).

Table 2 provides a detailed view of the clinical and pathological presentation of the study population. Analysis showed that 11.8% had a history of previous malignancies. However, those who refused surgery had a significantly higher proportion of individuals (20.6%) with previous malignancies when compared to the surgery group (11.8%, *p* < 0.001). Concerning the histological type, the papillary type encompassed 94.2% of the total study population and 94.5% of the refusal group, with no statistically significant difference in histological type between the surgery and refusal groups (*p* = 0.91).

Regarding tumor staging (T staging), 59.8% of the study population was classified as T1, with a lower percentage (48.9%) in the refusal group. Conversely, the refusal group had a higher percentage of T4 patients (13.5% vs. 3.2% in the surgery group), (*p* < 0.001). Nodal staging (N staging) showed that most patients were N0 (76.3%), with no significant difference between the surgery and refusal groups (*p* = 0.08). However, in metastatic staging (M staging), most patients were M0 (98.8%) but the refusal group had a higher percentage of M1 patients (8.8% vs. 1.2% in the surgery group, *p* < 0.001). Finally, when considering tumor extension, most tumors were localized (64.5%). Nevertheless, the refusal group had a higher proportion of patients with distant extension (15.7% vs. 2.4% in the surgery group), exhibiting a statistically significant difference (*p* < 0.001).

### 3.2. Determinants of Surgical Refusal in Cancer Patients

Table 3 elucidates the results of a multivariate logistic regression analysis, identifying key determinants that influenced the probability of surgical refusal in cancer patients. Patients aged 55 years or older were 1.57 times (95% CI: 1.12 to 2.19, *p* = 0.009) more likely to refuse surgery compared to their counterparts below 55 years of age. Males were more likely to refuse surgery (OR: 1.679, 95% CI: 1.215–2.320, *p* = 0.002) than females. In terms of racial categories, compared to White individuals, API (Asian or Pacific Islander) patients were 2.75 times more likely to refuse surgery (95% CI: 1.93 to 3.92, *p* < 0.001). However, the difference in surgical refusal between White and AI/AN (American Indian/Alaska Native) individuals was not statistically significant (OR = 1.12, 95% CI: 0.15 to 8.09, *p* = 0.91). Single individuals were more likely to refuse compared to married individuals (OR: 1.899, 95% CI: 1.321–2.729, *p* = 0.001) and widowed individuals were even more likely to refuse (OR: 4.268, 95% CI: 2.782–6.547, *p* < 0.001). 

Patients diagnosed with follicular type cancer were less likely to refuse than those with papillary type (OR: 0.316, 95% CI: 0.128–0.785, *p* = 0.013). Patients with T3/4 staging had higher odds of refusal than those with T1/2 staging (OR: 1.728, 95% CI: 1.204–2.478, *p* = 0.003). Metastatic patients (M1) were significantly more likely to refuse compared to nonmetastatic patients (M0) (OR: 6.402, 95% CI: 3.717–11.026, *p* < 0.001). In contrast, residency, income level, prior primary malignancy, and N staging did not significantly impact refusal.

### 3.3. Disease Outcomes

As depicted in Table 4, a considerable proportion of patients (43.3%) underwent radioactive iodine (RAI) therapy, with beam radiation, radioactive implants, and unspecified radiotherapy employed less frequently. In contrast, the refusal group showed a significant underutilization of RAI therapy, with only 0.9% opting for this treatment compared to the 43.4% in the surgery group (*p* < 0.001), which is sensible since RAI therapy generally cannot be performed before surgery. Most patients commenced their treatment within the first month (63.5%). However, treatment initiation was delayed to 4–6 months and ≥6 months more frequently in the refusal group. 

Regarding clinical outcomes, the occurrence of a second primary malignancy was not significantly different between the refusal group (7.8%) and those who underwent surgery (8.7%, *p* = 0.72). The overall mortality rate among the studied population was 8.8%, corresponding to 15,568 individuals. On the other hand, most of the total population, 91.2% or 160,904 individuals, were alive at the end of the study period (Table 4). 

### 3.4. Overall Survival Analysis

**Figure 1:**
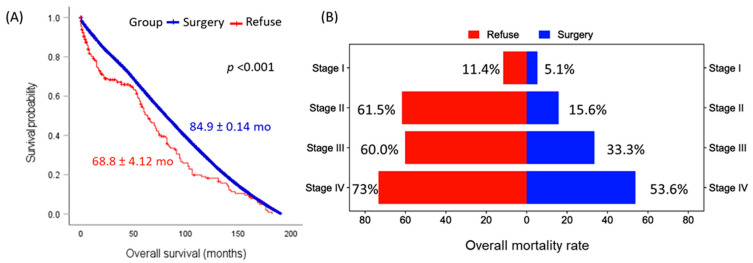
Comparison of overall survival in patients undergoing surgery versus those refusing surgery. (**A**) Kaplan–Meier survival curve comparing the surgery cohort (blue) and surgery-refusal group (red). Mean and standard-error survival times are presented. The log-rank test was employed for comparisons. (**B**) Bar chart illustrating the elevated mortality rate in patients who refused surgery compared to those who underwent surgery across all disease stages.

The analysis revealed a stark difference in survival status between the two groups, with a significantly more significant proportion of patients in the refusal group succumbing to their disease (31.9%) compared to the surgery group (8.8%, *p* < 0.001) (Table 4). The Kaplan–Meier survival curves corroborated this finding, indicating a statistically significant difference in overall survival between the surgery group (84.9 ± 0.14 months) and the refusal group (68.8 ± 4.12 months, *p* < 0.001), which is depicted in Figure 1.

After adjusting for potential confounders in the multivariate Cox regression analysis, refusal of surgery emerged as a prominent risk factor, leading to a 3.48-fold increased hazard of death (95% CI: 2.52–4.82, *p* < 0.001). Other significant contributors to overall mortality included age, sex, prior primary malignancy, and advanced disease stage, all identified in Table 5. 

**Figure 2:**
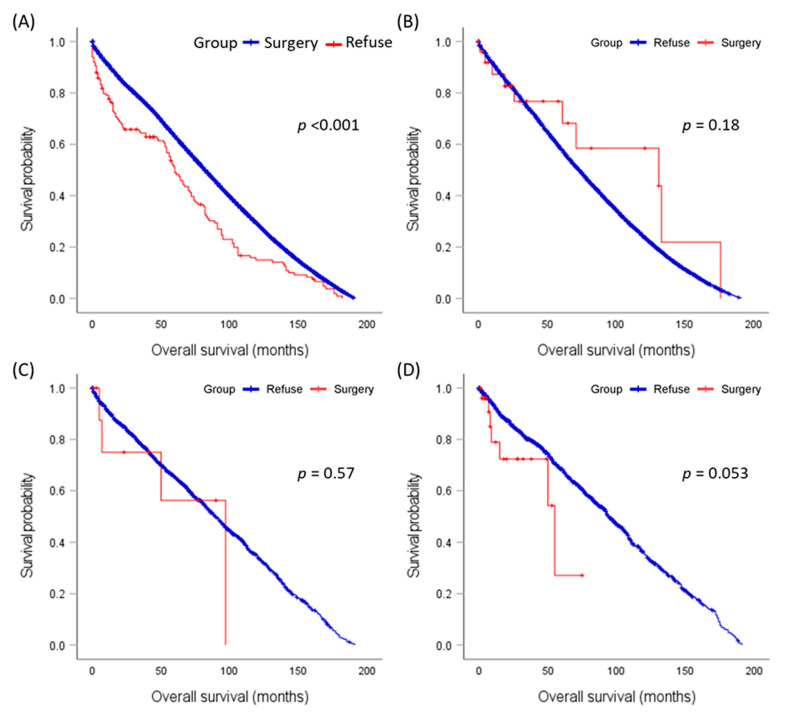
Stage-specific comparison of mean survival times in patients undergoing surgery versus those refusing surgery. (**A**) Stage I, (**B**) Stage II, (**C**) Stage III, (**D**) Stage IV. The log-rank test was employed for comparisons.

In a stage-specific comparison, the mean survival times varied across different stages of the disease and between groups. In the I stage, the mean survival time for the surgery group was 85.4 months, while it was significantly lower for the refusal group at 64.5 months. Similar trends were observed for other stages, with the surgery group consistently having higher mean survival times than the refusal group. Particularly in stage IV, the mean survival time for those who underwent surgery was more than double that of those who refused (94.9 months versus 46.7 months), as demonstrated in Figure 2.

### 3.5. Disease-Specific Survival Analysis

In the analysis of cause-specific mortality, most deaths in both groups, representing 85.9% (or 13,373 patients) of total deaths, were attributed to causes other than TC. Conversely, TC-specific mortality accounted for 14.1% of deaths, corresponding to 2195 individuals. Within these other causes, 18.4% (or 2865 patients) succumbed to nonthyroid malignancies and 67.5% (10,508 patients) died due to nonmalignant causes. When comparing TC-specific mortality between the two study groups, the refusal group showed a slightly higher rate of 18.0%, compared to the 14.1% observed in the surgery group. However, this difference was not statistically significant (*p* = 0.16). Interestingly, the refusal group had a significantly lower rate of death from non-thyroid malignancies (6.7% versus 18.5%, *p* = 0.003) but a higher rate of deaths from nonmalignant causes (75.3% versus 67.4%). 

In a subgroup analysis of 20,830 patients with prior cancer before thyroid cancer diagnosis, a death rate of 7.2% (or 1499 patients) was observed. Among the 13,597 individuals who developed a subsequent malignancy, the death rate from a subsequent malignancy was 10.0% (or 1366 individuals), similar in both groups, *p* = 0.99. In summary, these findings emphasize the crucial role of surgery in extending survival, despite most deaths being attributed to non-malignant diseases or non-thyroid malignancies.

As shown in Table S1, the predominant cause of non-TC mortality was death due to other malignancies, accounting for 2865 out of 13,373 total deaths, where 2855 patients had undergone surgery and ten refused. This was followed by heart and cerebrovascular diseases, causing 1577 and 391 deaths, respectively. Notably, a significant proportion of deaths in both the surgery and the refusal groups were attributed to unknown causes (*n* = 4378). The leading types of cancer-causing death among other malignancies were lung and bronchus (613 deaths), breast (299 deaths), pancreas (195 deaths), and colon (183 deaths) in that order, as shown in Table S2. 

The evaluation of risk factors for TC-specific mortality highlighted several significant determinants. Age greater than or equal to 55 years, male sex, and having a prior primary malignancy were all associated with increased hazard ratios in both univariable and multivariable analyses. Refusal of surgery dramatically increased the hazard of TC-specific death, even after adjusting for potential confounders (HR = 3.52, 95% CI = 1.93–6.42, *p* < 0.001). Furthermore, disease extension, both regional and distant, significantly contributed to TC-specific mortality, as shown in Table 6. Interestingly, delayed treatment (defined as treatment delay of 4 months or more) was not associated with TC-specific mortality after adjustment in multivariable analysis, with an HR of 0.96 (95% CI: 0.80–1.14, *p* = 0.64). While timely treatment is generally advised, slight delays may not substantially impact TC-specific mortality.

### 3.6. Impact of Delayed Surgery on Recurrence and Survival

**Figure 3:**
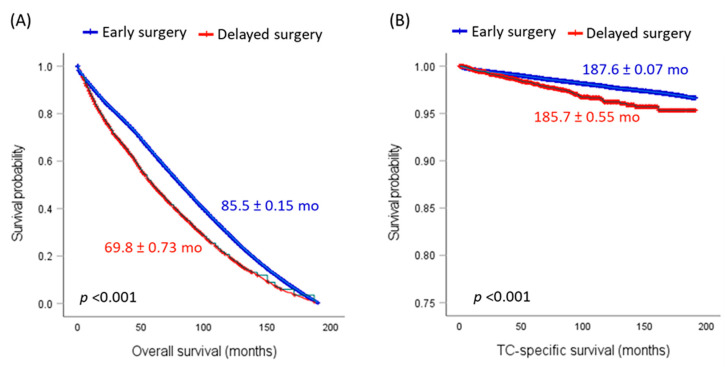
Survival analysis to compare cohorts according to the timing of the surgical procedure. (**A**) Overall survival. (**B**) Thyroid cancer (TC)-specific survival. Mean and standard error survival times are presented. The log-rank test was employed for comparisons.

An analysis of survival times and mortality rates was conducted among patients who underwent early surgery (<4 months, *n* = 140,149, 95.8%) and those who opted for delayed surgery (≥4 months, *n* = 5257, 3.6%). The results showcased a striking difference between the two groups. The mortality rate was notably higher in the delayed surgery group at 9.5%, compared to 7.1% in the early surgery group. Furthermore, the average survival times underscored this disparity further: 85.5 months (SE = 0.15) for early surgery versus 69.8 months (SE = 0.73) for delayed surgery (*p* < 0.001). Interestingly, the survival time for those who refused surgery was akin to the delayed surgery group, averaging 68.9 months (SE = 4.1), suggesting that delayed surgery might have equivalent consequences to altogether preceding the operation, as demonstrated in Figure 3.

However, when considering TC-specific survival analysis, the situation changes. This analysis included 132,315 patients from the early surgery group and 4857 from the delayed surgery group. The mortality rates due to TC were almost the same in both groups: 98.4% for early surgery and 98.0% for delayed surgery (*p* = 0.98). The survival times they have also exhibited a similar pattern, with 187.6 months (SE = 0.07) for early surgery and 185.7 months (SE = 0.55) for the delayed surgery group (*p* < 0.001). Both figures were markedly more prolonged than the survival time for the group that refused surgery (146.7 months, SE = 6.2). The data yield a significant insight: even though delaying surgery does affect overall survival, it does not seem to amplify the risk of death, specifically from TC. This suggests that the elevated mortality observed in the delayed surgery group could be attributed to other concurrent conditions or causes.

A comparison of causes of death between two groups of patients, those who underwent early surgery and those who had delayed surgery, is demonstrated in Table 7. The data indicate that delay in surgery is associated with a higher risk of death, both from TC and non-TC causes. Overall, delayed surgery is associated with an increased risk of death (HR = 1.37, 95% CI = 1.25–1.51, *p* < 0.001), and this increase is observed for both TC-related deaths (HR = 1.28, 95% CI = 1.05–1.58, *p* = 0.016) and non-TC related deaths (HR = 1.39, 95% CI = 1.25–1.55, *p* < 0.001).

Among non-TC causes of death, the most substantial increase in risk with delayed surgery is observed for other malignancies (HR = 1.89, *p* < 0.001), pneumonia and influenza (HR = 2.54, *p* = 0.001), and septicemia (HR = 2.25, *p* = 0.018). Interestingly, the risk of death from other causes such as diseases of the heart, cerebrovascular diseases, accidents and adverse effects, diabetes mellitus, chronic obstructive pulmonary disease, Alzheimer’s disease, nephritis, nephrotic syndrome and nephrosis, suicide and self-inflicted injury, chronic liver disease and cirrhosis, and other infectious and parasitic diseases including HIV did not significantly increase with delay in surgery (*p* > 0.05 in all cases). Hypertension without heart disease showed a trend towards increased risk with delayed surgery (HR = 2.05, *p* = 0.06), but this did not reach statistical significance, as shown in Table 7.

Regarding recurrence risk, univariate regression analysis showed both delayed surgery (HR = 0.85, 95% CI = 0.49–1.45, *p* = 0.55) and refusal of surgery (HR = 1.52, 95% CI = 0.21–10.8, *p* = 0.68) do not significantly affect the odds of recurrence as compared to early surgery. Similar findings were found in the multivariate regression model after adjusting for the effects of other factors. Our analysis showed that neither delayed surgery (HR = 0.65, 95% CI = 0.38–1.16, *p* = 0.11) nor refusal of surgery (1.41, 95% CI = 0.19–10.2, *p* = 0.73) significantly altered the odds of disease recurrence compared to early surgery.

## 4. Discussion

This study aimed to delve into the factors influencing patients’ decisions to refuse surgery for TC and to explore the potential consequences of surgical refusal. Using the SEER database, we analyzed a cohort of 176,472 TC patients, considering socioeconomic factors and clinicopathological characteristics to uncover intriguing trends that shed light on the decision-making process surrounding cancer surgical intervention.

Our findings revealed compelling insights regarding the factors contributing to patients’ decisions to refuse recommended surgery. Age was found to be a significant factor, with patients aged 55 years or older being more likely to refuse surgery. This finding aligns with previous studies that have highlighted age as a potential barrier to surgery in older patients. The reluctance to undergo surgery in older individuals may be attributed to concerns about the risks associated with anesthesia and postoperative complications, as well as personal preferences. It may also be attributed to their concern about their comorbidities possibly affecting their surgical outcomes or recovery. It is important to note that this refusal may be attributed to the patients’ concerns about these symptoms rather than the symptoms themselves. The latter is relatively unlikely considering that the coding system in the SEER database sorts patients having or not having surgery into different categories and that the present study extracted true refusals in which the physician explicitly recommended surgery, which the patients refused. With such physicians recommending surgery while having considered the patients’ age or comorbidities in the treatment plans, the surgery was medically justified. These findings emphasize the need to address age-related concerns and provide tailored counseling to older patients to ensure they have a comprehensive understanding of the potential benefits and risks of surgical intervention.

Gender also emerged as a determinant of surgical refusal, with males more likely to refuse surgery compared to females. This observation is consistent with existing literature, which has indicated that males may exhibit a higher level of apprehension and reluctance toward surgical interventions [14]. Further exploration is needed to understand the underlying factors contributing to this gender disparity and to develop targeted strategies to address these concerns. Exploring societal norms and cultural influences that shape patients’ treatment choices is crucial in this regard. Implementing targeted educational initiatives that address these factors could help bridge the gender gap and empower patients to make more informed decisions about their treatment options.

Additionally, our analysis unearthed disparities in surgical refusal among different racial groups. Specifically, Asian and Pacific Islander populations, as well as American Indian/Native American populations, demonstrated higher rates of surgical refusal. This highlights the need for a comprehensive approach to healthcare that considers cultural beliefs, language barriers, and access to information. By providing tailored education and improving cultural competency among healthcare providers, we can foster better patient–clinician communication and reduce disparities in treatment decisions [15].

Marital status played a notable role in surgical refusal, with single individuals and widowed individuals being more likely to refuse surgery compared to those who were married or in domestic partnerships. This observation suggests that a supportive partner or caregiver may influence patients’ decision-making. Single and widowed individuals may face unique challenges in managing the treatment process and may perceive a higher burden associated with surgery. Recognizing the importance of social support and providing additional resources for patients without a partner or caregiver can help address these concerns and support more informed treatment decisions.

Histological type, tumor staging, and metastatic status were also associated with surgical refusal. Patients with papillary type cancer, lower tumor staging, and nonmetastatic disease were more likely to undergo surgery, whereas those with follicular type cancer and advanced disease stages demonstrated a higher propensity to refuse surgery. These findings emphasize the need for tailored communication strategies and counseling to address patient concerns and misconceptions about disease severity, treatment options, and prognosis. Clear and accurate information about the benefits of surgery, even in advanced stages of the disease, is essential to ensure patients make well-informed decisions.

Regarding recurrence risk, our analysis indicated that neither delayed surgery nor refusal of surgery significantly affected the odds of disease recurrence compared to early surgery. These findings suggest that delaying or refusing surgery may not significantly impact the likelihood of disease recurrence. However, further studies with extended follow-up periods and larger sample sizes are warranted to confirm these findings.

Regarding survival outcomes, our analysis confirmed the significant impact of surgery on overall mortality. Surgery refusal was identified as a prominent risk factor associated with a 3.48-fold increased hazard of death. This finding is consistent with prior research emphasizing the importance of surgical intervention in improving survival rates, minimizing disease progression, and reducing mortality risks associated with TC.

To further corroborate these findings, we generated two literature-review tables summarizing the outcomes of surgery refusal in TC patients from multiple databases. The studies summarized in Table 8 repeatedly showed higher overall survival and lower disease-specific mortality in surgery cohorts compared to no-surgery cohorts. Studies focused on outcomes in thyroid-specific surgery (PTC, ATC, and MTC) demonstrate higher overall survival and lower disease-specific mortality in surgery cohorts compared to no-surgery cohorts. Delaying or refusing thyroid surgery was associated with lower overall rates of survival. The data underscore the critical role of surgery in managing thyroid cancer patients and emphasize the need for timely intervention. 

Studies summarized in Table 9 show results for non-TC patients with other types of cancer and the findings once again indicate worse outcomes for those refusing surgery. Surgery refusal in nonthyroid cancers was consistently associated with lower overall survival than groups that underwent surgery (prostate, pituitary, stomach, oral, rectal, colorectal, pancreatic, ovarian, liver, and breast). Cancer-specific mortality and overall survival were the primary outcomes examined. Taken together, this data underscores the critical role of timely surgical intervention when indicated in the management of TC patients.

Furthermore, we explored the impact of surgical intervention timing on survival outcomes. Our results solidify the importance of early surgical intervention in managing TC. The study findings indicate that, while delayed surgery for TC does not increase the risk of death specifically from TC itself, it is associated with an elevated risk of mortality from other causes. The increased risk was particularly notable for other malignancies, pneumonia and influenza, and septicemia. A 2023 study using the SEER–Medicare linked database demonstrated that patients who delayed surgery for more than 180 days had almost four times the estimated DSS mortality [17]. It is essential to consider various external factors, including patient factors (e.g., comorbidities, lifestyle choices, socioeconomic status, and treatment preferences), healthcare-system factors (e.g., waiting times, access to specialized care, healthcare infrastructure, and surgical expertise), and clinical factors (e.g., disease progression and treatment planning), which can influence patient outcomes and mortality rates. Therefore, a holistic approach to patient care that addresses these multifaceted factors is crucial for improving overall survival rates. The analysis comparing causes of death between the early surgery and delayed surgery groups highlights the importance of timely intervention, effective referral systems, and streamlined treatment pathways in TC patients to minimize the risk of mortality from both TC and other comorbidities [1]. These observations highlight the significance of vigilant monitoring and management of other health conditions in these patients and can guide informed decisions about the timing of surgery, balancing the urgency of the procedure against the risks associated with delay.

The present study utilized a robust sample size and a reliable SEER database, enhancing the generalizability and validity of the findings. However, it is essential to acknowledge the limitations inherent in retrospective data analysis, which may carry the risk of information and selection biases. Future studies should incorporate qualitative data, allowing for a nuanced exploration of patient beliefs, preferences, and values to understand patient decision-making better. This qualitative approach will provide novel insights into the complex factors influencing surgical refusal and guide the development of patient-centered interventions [28]. It can also assist physicians in empathizing with the patients and addressing their concerns. Additionally, information comparing the quality of life of TC patients who agree to surgery to those who delay or refuse surgery is not available in the SEER database. Assessing this may be helpful in patients with older age or comorbidities; considering that the present study analyzed true refusals when surgery was explicitly recommended by the physicians, it may be possible that these patients may experience more severe symptoms later in life which may be due to either TC from surgery refusal or these comorbidities. As such, assessing the quality of life and comparing it to the TC-specific mortality rates may provide further insight.

## 5. Conclusions

Our study provides novel insights into the determinants and consequences of surgical refusal in TC patients. By identifying gender disparities, racial and geographic variations, and the impact of surgical timing on survival outcomes, we underscore the importance of informed decision-making and tailored patient care. Efforts should be directed toward addressing sociodemographic and cultural disparities and fostering a comprehensive approach that considers the unique circumstances of each patient to optimize outcomes. The present study should also motivate physicians to encourage and continue educating their patients on the importance of surgical intervention when medically justified, even upon the patient’s instant refusal. The emphasis on continuous encouragement and education has useful implications for daily clinical practice. Future research should continue to explore the qualitative aspects of patient decision-making to enhance our understanding further and refine patient-care strategies. 

## Figures and Tables

**Table 1:** Demographic and socioeconomic characteristics of the study population.

Characteristics	Levels	Total Patients (*n* = 176,472)	Surgery Performed (*n* = 176,002)	Patient Refused (*n* = 470)	*p*-Value
Total number		176,472	176,002 (99.7)	470 (0.3)	
Age (years)	Median (IQR)	49.0 (38–60)	49.0 (38–60)	59.0 (44–74)	**<0.001**
	<55 years	110,252 (62.5)	110,055 (62.5)	197 (41.9)	**<0.001**
	≥55 years	66,220 (37.5)	65,947 (37.5)	273 (58.1)	
Gender	Female	134,960 (76.5)	134,637 (76.5)	323 (68.7)	**<0.001**
	Male	41,512 (23.5)	41,365 (23.5)	147 (31.3)	
Race	White	143,085 (82.1)	142,752 (82.1)	333 (72.1)	**<0.001**
	Black	11,063 (6.3)	11,031 (6.3)	32 (6.9)	
	API	18,998 (10.9)	18,908 (10.9)	90 (19.5)	
	AI/AN	1168 (0.7)	1161 (0.7)	7 (1.5)	
Ethnicity	Not Hispanic/Latino	147,128 (83.4)	146,740 (83.4)	388 (82.6)	0.68
	Hispanic/Latino	29,344 (16.6)	29,262 (16.6)	82 (17.4)	
Marital status	Married/domestic partner	108,619 (64.8)	108,410 (64.9)	209 (48.9)	**<0.001**
	Single	36,539 (21.8)	36,431 (21.8)	108 (25.3)	
	Separated/divorced	13,900 (8.3)	13,858 (8.3)	42 (9.8)	
	Widowed	8485 (5.1)	8417 (5.0)	68 (15.9)	
Metropolitan	Metropolitan > 1 M pop	106,097 (60.2)	105,788 (60.2)	309 (66.2)	0.11
	Metropolitan > 250 K–1 M	39,729 (22.5)	39,643 (22.5)	86 (18.4)	
	Metropolitan of <250 K	13,149 (7.5)	13,118 (7.5)	31 (6.6)	
	Nonmetropolitan adj to a Metropolitan	9925 (5.6)	9903 (5.6)	22 (4.7)	
	Nonmetropolitan not adj to Metropolitan	7370 (4.2)	7351 (4.2)	19 (4.1)	
Household annual income	USD 75,000+	60,727 (34.4)	60,547 (34.4)	180 (38.3)	0.33
	USD 70,000–USD 74,999	16,114 (9.1)	16,069 (9.1)	45 (9.6)	
	USD 65,000–USD 69,999	27,760 (15.7)	27,681 (15.7)	79 (16.8)	
	USD 60,000–USD 64,999	28,092 (15.9)	28,016 (15.9)	76 (16.2)	
	USD 55,000–USD 59,999	11,380 (6.4)	11,360 (6.5)	20 (4.3)	
	USD 50,000–USD 54,999	13,498 (7.6)	13,466 (7.7)	32 (6.8)	
	USD 45,000–USD 49,999	7801 (4.4)	7783 (4.4)	18 (3.8)	
	USD 40,000–USD 44,999	5760 (3.3)	5748 (3.3)	12 (2.6)	
	USD 35,000–USD 39,999	3163 (1.8)	3158 (1.8)	5 (1.1)	
	<USD 35,000	2163 (1.2)	2160 (1.2)	3 (0.6)	

Data are presented as number and percentage or median and interquartile range (IQR). Two-sided Chi-Square or Mann–Whitney U tests were used. Bold values indicate statistical significance at *p*-value < 0.05. API: Asian or Pacific Islander, AI/AN: Am. Indian/Alaska Native, M: million.

**Table 2:** Clinical and pathological presentation of the study population.

Characteristics	Levels	Total (*n* = 176,472)	Surgery Performed (*n* = 176,002)	Patient Refused (*n* = 470)	*p*-Value
Previous malignancies	No	155,637 (88.2)	155,264 (88.2)	373 (79.4)	**<0.001**
	Yes	20,830 (11.8)	20,733 (11.8)	97 (20.6)	
Histological Type	Papillary	166,311 (94.2)	165,867 (94.2)	444 (94.5)	0.91
	Follicular	10,161 (5.8)	10,135 (5.8)	26 (5.5)	
T staging	T1	90,418 (59.8)	90,309 (59.8)	109 (48.9)	**<0.001**
	T2	26,415 (17.5)	26,358 (17.5)	57 (25.6)	
	T3	29,482 (19.5)	29,455 (19.5)	27 (12.1)	
	T4	4859 (3.2)	4829 (3.2)	30 (13.5)	
N staging	N0	128,380 (76.3)	128,105 (76.3)	275 (72.4)	0.08
	N1	39,855 (23.7)	39,750 (23.7)	105 (27.6)	
M staging	M0	174,068 (98.8)	173,641 (98.8)	427 (91.2)	**<0.001**
	M1	2161 (1.2)	2120 (1.2)	41 (8.8)	
Extension	Localized	112,842 (64.5)	112,594 (64.5)	248 (62.6)	**<0.001**
	Regional	57,949 (33.1)	57,863 (33.1)	86 (21.7)	
	Distant	4273 (2.4)	4211 (2.4)	62 (15.7)	

Data are presented as numbers and percentages. A two-sided Chi-Square test was used. Bold values indicate significance at a *p*-value < 0.05.

**Table 3:** Factors influencing patients’ refusal of recommended cancer surgery.

Determinant of Surgical Refusal	Odds Ratio	Lower Limit	Upper Limit	*p*-Value
Age: ≥55 vs. <55 years old	1.570	1.122	2.197	**0.009**
Sex: Male vs. Female	1.679	1.215	2.320	**0.002**
Race: White vs. Black	1.770	1.069	2.929	**0.026**
Race: White vs. API	2.754	1.931	3.928	**<0.001**
Race: White vs. AI/AN	1.119	0.155	8.096	0.91
Single vs. Married	1.899	1.321	2.729	**0.001**
Separated/divorced vs. Married	1.309	0.741	2.314	0.35
Widowed vs. Married	4.268	2.782	6.547	**<0.001**
Residency: Urban vs. Rural	1.271	0.714	2.264	0.42
Income ≥USD 75,000 vs. <USD 75,000	1.038	0.761	1.417	0.81
Prior primary malignancy vs. None	1.169	0.787	1.735	0.43
Histopathology: Follicular vs. Papillary	0.316	0.128	0.785	**0.013**
T staging: T3/4 vs. T1/2	1.728	1.204	2.478	**0.003**
N staging: N1 vs. N0	0.782	0.538	1.137	0.19
M staging: M1 vs. M0	6.402	3.717	11.026	**<0.001**

Multivariate logistic regression analysis was employed (*n* = 146,238 after removing missing). The odds ratio (OR) and 95% confidence interval was reported. Bold values indicate significance at a *p*-value < 0.05.

**Table 4:** Treatment modalities and disease outcomes in thyroid cancer patients.

Characteristics	Levels	Total (*n* = 176,472)	Surgery Performed (*n* = 176,002)	Patient Refused (*n* = 470)	*p*-Value
Management					
Radiotherapy	RAI	76,331 (43.3)	76,327 (43.4)	4 (0.9)	**<0.001**
	Beam radiation	1863 (1.1)	1845 (1.0)	18 (3.8)	
	Radioactive implants	1278 (0.7)	1278 (0.7)	0 (0.0)	
	Unspecified radiotherapy	981 (0.6)	981 (0.6)	1 (0.0)	
Time to treatment	<1 month	111,162 (63.5)	111,109 (63.5)	53 (60.2)	**<0.001**
	1–3 months	57,943 (33.1)	57,924 (33.1)	19 (21.6)	
	4–6 months	4562 (2.6)	4552 (2.6)	10 (11.4)	
	≥6 months	1429 (0.8)	1423 (0.8)	6 (6.8)	
Clinical outcomes					
Second primary malignancy	Positive	13,597 (8.7)	13,568 (8.7)	29 (7.8)	0.72
Survival status	Died	15,568 (8.8)	15,418 (8.8)	150 (31.9)	**<0.001**

Data are presented as numbers and percentages or median and interquartile range (IQR). Two-sided Chi-Square and Mann–Whitney U tests were used. Bold values indicate significance at a *p*-value < 0.05.

**Table 5:** Predictor risk factors for overall mortality.

Risk Factors for Mortality	Frequency	HR (Univariable)	HR (Multivariable)
Age: ≥55 vs. <55 years old	69,710 (38.2)	7.74 (7.46–8.02, *p* < 0.001)	6.20 (5.96–6.45, *p* < 0.001)
Sex: Male vs. Female	43,599 (23.9)	2.22 (2.16–2.29, *p* < 0.001)	1.55 (1.50–1.60, *p* < 0.001)
Ethnicity: Hispanic/Latino vs. None	30,509 (16.7)	0.78 (0.74–0.82, *p* < 0.001)	0.95 (0.91–1.00, *p* = 0.07)
Household income: >USD 75,000 vs. <USD 75,000	62,906 (34.4)	0.79 (0.77–0.82, *p* < 0.001)	0.84 (0.81–0.87, *p* < 0.001)
Residency: Urban vs. Rural	164,605 (90.2)	0.71 (0.68–0.75, *p* < 0.001)	0.79 (0.75–0.82, *p* < 0.001)
Histopathology: Papillary vs. Follicular	10,662 (5.8)	1.63 (1.55–1.71, *p* < 0.001)	1.31 (1.24–1.38, *p* < 0.001)
Prior primary malignancy vs. None	22,555 (12.4)	3.98 (3.85–4.10, *p* < 0.001)	2.27 (2.19–2.35, *p* < 0.001)
Surgery: Patient refused vs. Operated	470 (0.3)	5.98 (5.09–7.02, *p* < 0.001)	3.48 (2.52–4.82, *p* < 0.001)
Extension: Regional vs. Localized	58,810 (32.8)	1.28 (1.24–1.32, *p* < 0.001)	1.39 (1.34–1.44, *p* < 0.001)
Extension: Distant vs. Localized	5102 (2.8)	6.80 (6.48–7.13, *p* < 0.001)	4.75 (4.48–5.03, *p* < 0.001)
Delayed treatment: ≥4 vs. <4 months	6096 (3.5)	1.64 (1.52–1.78, *p* < 0.001)	1.26 (1.16–1.36, *p* < 0.001)

Cox regression hazards proportional test was used. Hazards ratios and 95% confidence intervals (CI) were reported. Statistical significance was set at *p*-value < 0.05.

**Table 6:** Predictor risk factors for thyroid cancer-specific mortality.

Risk Factors for Disease-Specific Mortality	Frequency	HR (Univariable)	HR (Multivariable)
Age: ≥55 vs. <55 years old	56,586 (34.5)	9.11 (8.36–9.92, *p* < 0.001)	8.43 (7.71–9.22, *p* < 0.001)
Sex: Male vs. Female	36,915 (22.5)	2.72 (2.53–2.91, *p* < 0.001)	1.61 (1.50–1.73, *p* < 0.001)
Ethnicity: Hispanic/Latino vs. None	28,060 (17.1)	1.11 (1.01–1.22, *p* = 0.028)	1.21 (1.10–1.33, *p* < 0.001)
Household income: >USD 75,000 vs. <USD 75,000	56,955 (34.8)	0.85 (0.79–0.91, *p* < 0.001)	0.91 (0.85–0.99, *p* = 0.024)
Residency: Urban vs. Rural	148,123 (90.5)	0.76 (0.69–0.85, *p* < 0.001)	0.80 (0.71–0.89, *p* < 0.001)
Histopathology: Papillary vs. Follicular	9156 (5.6)	2.31 (2.08–2.56, *p* < 0.001)	1.58 (1.42–1.76, *p* < 0.001)
Prior primary malignancy vs. None	16,955 (10.3)	2.63 (2.42–2.87, *p* < 0.001)	1.50 (1.37–1.63, *p* < 0.001)
Surgery: Patient refused vs. Operated	470 (0.3)	17.3 (9.55–31.4, *p* < 0.001)	3.52 (1.93–6.42, *p* < 0.001)
Extension: Regional vs. Localized	53,950 (33.2)	4.07 (3.73–4.44, *p* < 0.001)	4.44 (4.07–4.85, *p* < 0.001)
Extension: Distant vs. Localized	3715 (2.3)	39.9 (36.3–43.9, *p* < 0.001)	33.8 (30.7–37.4, *p* < 0.001)
Delayed treatment: ≥4 vs. <4 months	5483 (3.4)	1.52 (1.28–1.81, *p* < 0.001)	0.96 (0.80–1.14, *p* = 0.64)

Cox regression hazards proportional test was used. Hazards ratios and 95% confidence intervals (CI) were reported. Statistical significance was set at *p*-value < 0.05.

**Table 7:** Effect of delayed surgery on survival.

Cause of Death	Early Surgery (*n* = 140,149)	Delayed Surgery (*n* = 5257)	HR (95% CI)	*p*-Value
Alive	130,253	4760	Reference	
Death	9896	497	1.37 (1.25–1.51)	**<0.001**
Thyroid cancer	2062	97	1.28 (1.05–1.58)	**0.016**
Nonthyroid cancer	7834	400	1.39 (1.25–1.55)	**<0.001**
Other malignancies	2670	185	1.89 (1.62–2.21)	**<0.001**
Diseases of Heart	1511	60	1.08 (0.83–1.4)	0.53
Cerebrovascular Diseases	373	15	1.1 (0.65–1.84)	0.71
Pneumonia and Influenza	140	13	2.54 (1.43–4.48)	**0.001**
Accidents and Adverse Effects	329	12	0.99 (0.56–1.77)	**0.007**
Diabetes Mellitus	218	10	1.25 (0.66–2.36)	0.7
Septicemia	109	9	2.25 (1.14–4.46)	**0.018**
Chronic Obstructive Pulmonary Disease	299	8	0.73 (0.36–1.47)	0.38
Alzheimer’s (ICD-9 and 10 only)	178	7	1.07 (0.51–2.29)	0.84
Hypertension without Heart Disease	93	7	2.05 (0.95–4.44)	0.06
Nephritis, Nephrotic Syndrome, and Nephrosis	188	5	0.73 (0.29–1.76)	0.48
Suicide and Self-Inflicted Injury	90	3	0.91 (0.28–2.88)	0.87
Chronic Liver Disease and Cirrhosis	72	2	0.76 (0.18–3.09)	0.7
Other Infectious and Parasitic Diseases, including HIV	75	2	0.79 (0.18–2.97)	0.66

Univariate Cox regression analysis was employed. Hazards ratio (HR) and 95% confidence intervals (CI) were reported. HR > 1 suggests an increased risk with delayed surgery compared to early surgery, and HR < 1 suggests a decreased risk with delayed surgery. Bold values indicate significance at a *p*-value < 0.05.

**Table 8:** Literature review of surgery-refusal outcomes in thyroid cancer.

Study Characteristics		Data Source	Study Population	Sample Size			Outcomes	
Author, Year (Ref.)	Country			Total	Surgery	No Surgery	Cancer-Directed Surgery Cohort	No Surgery Cohort
Wu, 2023 [16]	USA	Institutional (2000–2021)	ATC	97	44	53	Higher OS	Lower OS
Chaves, 2022 [17]	USA	SEER (1999–2018)	Medicare beneficiaries, PTC	8170	8170	-	Surgery (<90 days): high OS, DSS Surgery (>180 days): lower OS, DSS	
Sahli, 2021 [18]	USA	SEER	MTC (Exclude lobectomy)	1367	1301	66	Lower disease-specific mortality	Higher disease-specific mortality
Zhou, 2020 [19]	CHN	SEER (1973–2015)	>85 years old, PTC	1196	871	325	Higher OS	Lower OS
Van Gerwen, 2020 [1]	USA	SEER (1988–2015)	Localized and regional, PTC	45,136	44,990	146	Higher DSS time	Lower DSS times *
Maniakas, 2020 [20]	USA	Tertiary Care Center (2000–2019)	ATC	479	55	424	1-year survival; 94%	1-year survival; 52%
Corrigan, 2019 [21]	USA	Institutional (1990–2015)	ATC	28	19	9	Higher OS	Lower OS
Megwalu, 2017 [6]	USA	SEER (1988–2009)	>65 years old, PTC	2323	-	-	5-year survival rate; 91%	5-year survival rate; 23%
Pierie, 2002 [22]	USA	Tertiary Care Center (1969–1999)	ATC	67	44	23	Higher 6-month, 1- and 3-year survival rates	Lower 6-month, 1 and 3-year survival rates

USA: United States of America, ATC: anaplastic thyroid cancer, OS: overall survival, SEER: The Surveillance, Epidemiology, and End Results, NCDB: National Cancer Database, PTC: papillary thyroid carcinoma, DSS: Disease-specific survival. * Surgery recommended but refused by the patient.

**Table 9:** Literature review of surgery-refusal outcomes in other cancers.

Study Characteristics		Data Source	Study Population	Sample Size			Outcomes	
Author, Year (Ref.)	Country			Total	Surgery	No Surgery	Cancer-Directed Surgery Cohort	No Surgery Cohort
Chen, 2023 [23]	China	Institutional (2010–2019)	Localized and advanced Prostate cancer	19,729	6339	13,390	Reduced rates of cancer-specific and overall mortality	Increased rates of cancer-specific and overall mortality
Birkenbeuel, 2022 [4]	USA	NCDB (2004–2015)	Pituitary macroadenoma	34,226	33,946	280	Increased OS	Reduced OS *
Sun, 2022 [9]	China	SEER (2010–2015)	Stage IV gastric cancer	6284, matched 864	432	432	Prolonged median survival time	Reduced median survival time
Silva, 2021 [24]	Brazil	Hospital Haroldo Juaçaba (2000–2014)	Oral Squamous Cell Carcinoma	934	-	-	Higher OS	Lower OS
Coffman, 2021 [3]	USA	NCDB (2004–2015)	Rectal adenocarcinoma	55,704	54,266	1438	Median survival time 84.4 months	Median survival time 48.5 months *
Delisle, 2020 [8]	USA	SEER (2004–2015)	Colorectal	153,698	152,731	983	Lower disease-specific mortality	Higher disease-specific mortality
Coffman, 2019 [25]	USA	NCDB (2004–2013)	Nonmetastatic pancreatic adenocarcinoma	48,902	47,107	1795	Higher median survival times	Lower median survival times *
May 2017 [26]	Canada	Tom Baker Cancer Center (not provided)	Stage IIIC or IV serous ovarian carcinoma	303	142	161	5-year survival; 39%	5-year survival; 27%
Wang, 2010 [7]	USA	SEER (not provided)	Hepatocellular carcinoma	4373	4231	142	Lower mortality risk	2.5-fold increased mortality risk *
Verkooijen, 2005 [27]	Switzerland	Geneva Cancer Database (1975–2000)	<80 years old, nonmetastatic	5339	5269	70	Lower mortality risk	2.1-fold increased mortality risk *

USA: United States of America, OS: overall survival, SEER: The Surveillance, Epidemiology, and End Results, NCDB: National Cancer Database, * Surgery recommended but refused by the patient.

## Data Availability

Data needed is available within the manuscript. The SEER database is available online.

## Supplementary Materials

The following are available online at https://www.mdpi.com/article/10.3390/cancers15143699/s1, Table S1: Mortality due to nonthyroid cancer causes. Table S2: Common primary malignancies lead to nonthyroid cancer mortality.
