# Supplementary material for: Navigating Choices: Determinants and Outcomes of Surgery Refusal in Thyroid Cancer Patients Using SEER Data
Source: Cancers (Basel). 2023 Jul 20;15(14):3699. doi: 10.3390/cancers15143699 (PMC10378250; doi:10.3390/cancers15143699)
Supplement: Supplementary file 1 [file cancers-15-03699-s001.zip › cancers-2490144-supplementary.pdf]

Navigating Choices: Determinants and Outcomes of Surgery Refusal in Thyroid Cancer Patients Using SEER Data

Supplementary Table S1. Mortality due to non-thyroid cancer causes.

**Causes of death**	**Surgery (*n* = 13250)**	**Refusal (*n* = 123)**	**Total (*n* = 13373)**
Other malignancies	2855	10	2865
Diseases of Heart	1571	6	1577
Cerebrovascular Diseases	388	3	391
Accidents and Adverse Effects	341	1	342
Chronic Obstructive Pulmonary Disease and Allied Cond	307	1	308
Diabetes Mellitus	228	1	229
Nephritis, Nephrotic Syndrome and Nephrosis	193	1	194
Alzheimer's (ICD-9 and 10 only)	185	0	185
Pneumonia and Influenza	153	0	153
Septicemia	118	0	118
Hypertension without Heart Disease	100	0	100
Suicide and Self-Inflicted Injury	93	0	93
Other Infectious and Parasitic Diseases, including HIV	77	1	78
Chronic Liver Disease and Cirrhosis	74	0	74
Aortic Aneurysm and Dissection	35	0	35
Homicide and Legal Intervention	20	0	20
Other Diseases of Arteries, Arterioles, Capillaries	19	0	19
Atherosclerosis	15	0	15
Congenital Anomalies	10	0	10
Complications of Pregnancy, Childbirth, Puerperium	3	0	3
Unknown	4306	72	4378

Supplementary Table S2. Common primary malignancies leading to non-thyroid cancer mortality.

**Cancer type**	**Surgery (*n* = 2855)**	**Refusal (*n* = 10)**	**Total (*n* = 2865)**
Lung and Bronchus	609	4	613
Breast	297	2	299
Pancreas	194	1	195
Colon excluding Rectum	183	0	183
Non-Hodgkin Lymphoma	127	0	127
Ovary	109	0	109
Brain and Other Nervous System	103	0	103
Kidney and renal pelvis	90	0	90
Melanoma of the Skin	88	1	89
Myeloma	84	0	84
Liver	72	0	72
Acute Myeloid Leukemia	70	0	70
Prostate	65	0	65
Stomach	57	0	57
